# Supplementary material for: Negative Relationship between Post-Treatment Stromal Tumor-Infiltrating Lymphocyte (TIL) and Survival in Triple-Negative Breast Cancer Patients Treated with Dose-Dense Dose-Intense NeoAdjuvant Chemotherapy
Source: Cancers (Basel). 2022 Mar 4;14(5):1331. doi: 10.3390/cancers14051331 (PMC8909288; doi:10.3390/cancers14051331)
Supplement: Supplementary file 1 [file cancers-14-01331-s001.zip › cancers-1604825-Figure S1 and Tables S1-S6.pdf]

cancers MDPI

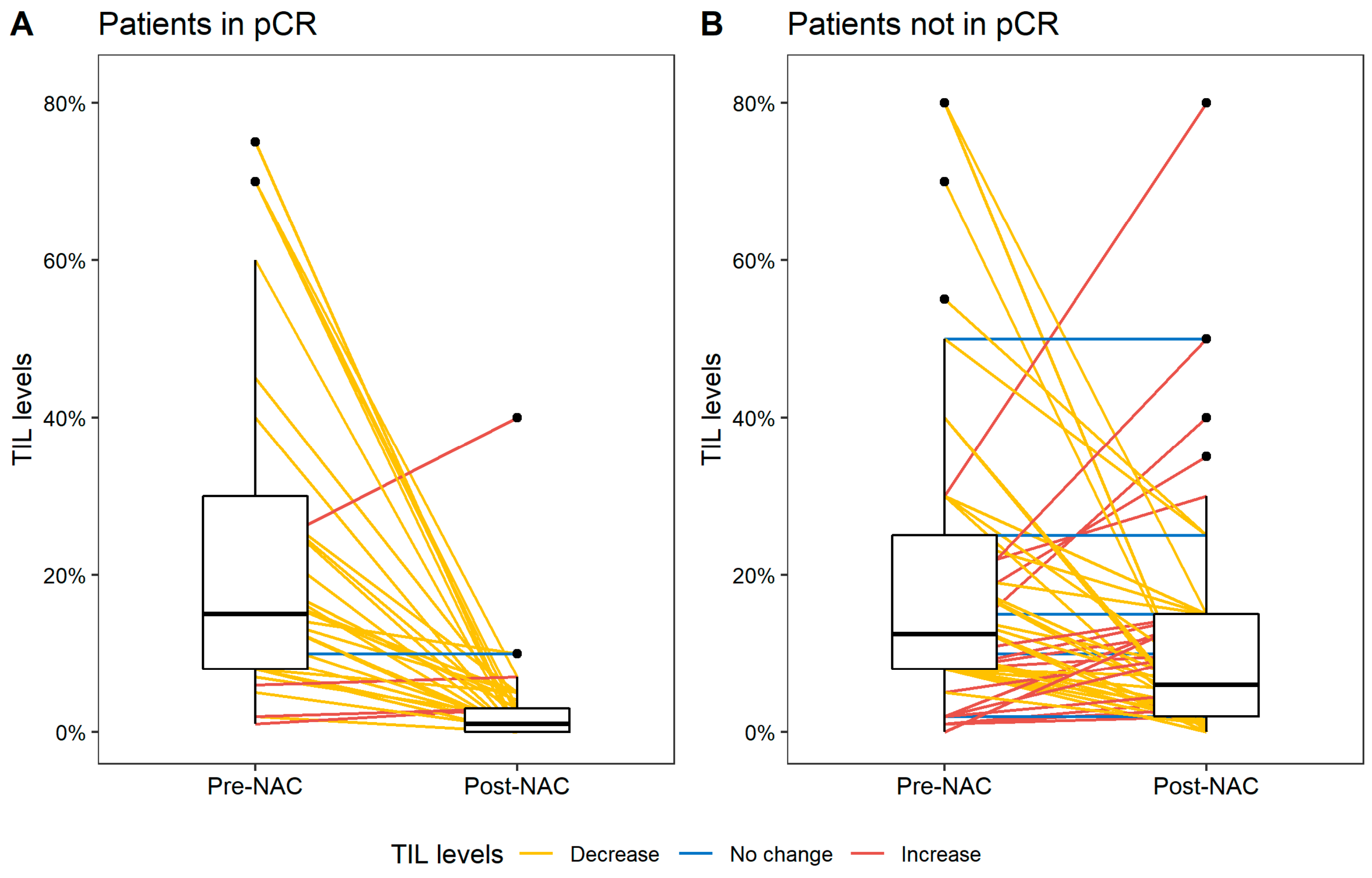

Figure S1. Change in TIL levels after NAC, according to the response to NAC, A: Change in TIL levels for the patients that achieved pCR, B: Change in TIL levels for the patients that did not achieve pCR.

Table S1. Predictive factors associated with pre-NAC TIL levels in univariate analyses.

	Levels	coefficient	[95% CI]	P-value	
	**Pre-NAC parameters**				
**Age (class)**	<45				
	≥45	-0.3	[-0.73 ; 0.13]	0.173	
**Pregnancies**	0				
	1-3	0.21	[-0.23 ; 0.65]	0.347	
	>3	-0.11	[-0.7 ; 0.48]	0.709	
**Menopausal status**	premenopausal				
	postmenopausal	-0.15	[-0.54 ; 0.23]	0.43	
**Family history**	0				
	1	0.11	[-0.39 ; 0.61]	0.67	
	≥2	0.47	[-0.16 ; 1.1]	0.14	
**Clinical tumor stage**	T1-T2				
	T3	-0.26	[-0.64 ; 0.13]	0.186	
**Clinical nodal status**	N-				
	N+	0.086	[-0.3 ; 0.47]	0.658	
**Tumor grade**	2				
	3	0.25	[-0.25 ; 0.74]	0.33	
**LVI**	Absence				
	Presence	0.13	[-0.39 ; 0.64]	0.63	
**SUV (continuous)**[a]				**0.025**	*
**Chemotherapy regimen**	SIM-1				

	SIM-2	-0.13	[-0.58 ; 0.32]	0.563

[a]continuous variable modelized with a degree 3 polynomial (coefficients and IC : -0.3 [-0.62 ; 0.025] ; 0.024 [0.0013 ; 0.047] ; -0.0005 [-0.00098 ; -0.0000018]).

Table S2. Predictive factors associated with SUV relative variation.

		Univariate analyses				Multivariate analysis			
**Parameters**	**Levels**	**coefficient**	**IC 95%**	**p-value**		**coefficient**	**IC 95%**	**p-value**	
	**Pre-NAC parameters**								
**Age (class)**	<45								
	≥45	-1.4	[-13 ; 9.7]	0.804					
**Pregnancies**	0								
	1-3	-4.2	[-16 ; 7.4]	0.476					
	>3	3.1	[-13 ; 20]	0.709					
**Menopausal status**	premenopausal								
	postmenopausal	1.8	[-8.5 ; 12]	0.728					
**Family history**	0								
	1	3.7	[-8.9 ; 16.2]	0.57					
	≥2	15.7	[-0.85 ; 32.3]	0.063	.				
**Clinical tumor stage**	**T1-T2**					**12.3**	**[3.3 ; 21.4]**	**0.008**	**
	**T3**	**16.4**	**[6.8 ; 26]**	**<0.001**	***				
**Clinical nodal status**	N-								
	N+	4.9	[-5.2 ; 15]	0.337					
**Tumor grade**	**2**								
	**3**	**-25.3**	**[-39 ; -12]**	**<0.001**	***	**-22.3**	**[-34.8 ; -9.8]**	**0.0007**	***
**LVI**	Absence								
	Presence	7	[-6.1 ; 20.1]	0.29					
**SUV**		-0.66	[-1.6 ; 0.22]	0.14					
**TIL levels (continuous)[a]**		**-0.32**	**[-0.56 ; -0.079]**	**0.010**	**			**0.042**	*

[a]continuous variable modelized with a degree 3 polynomial in the multivariate analysis (coefficients and IC : -59.1 [-111 ; -7.1] ; 19.4 [-29.8 ; 68.5] ; -50.6 [-100 ; -1.1]).

Table S3. Predictive factors associated with TIL levels absolute variation.

**Parameters**	**Levels**	**coefficient**	**[95% CI]**	**P-value**	
	**Pre-NAC parameters**				
**Age (class)**	<45				
	≥45	-3.2	[-14 ; 7.5]	0.555	
**Pregnancies**	0				
	1-3	-6.6	[-18 ; 4.3]	0.234	
	>3	1.6	[-13 ; 16]	0.825	
**Menopausal status**	premenopausal				
	postmenopausal	-2.2	[-12 ; 7.4]	0.647	
**Family history**	0				
	1	1.7	[-11.3 ; 14.7]	0.79	
	≥2	6.6	[-9.4 ; 22.6]	0.41	
**Clinical tumor stage**	**T1-T2**				
	**T3**	**9.4**	**[-0.0091 ; 19]**	**0.05**	.
**Clinical nodal status**	N-				
	N+	1.9	[-7.7 ; 11]	0.7	
**Tumor grade**	2				
	3	-0.83	[-13 ; 12]	0.895	
**LVI**	Absence				
	Presence	9.2	[-4.4 ; 22.7]	0.18	
**SUV**		**-0.93**	**[-1.7 ; -0.11]**	**0.027**	*
**TIL levels (continuous)[a]**				**<0.0001**	***
**Chemotherapy regimen**	SIM-1				

	SIM-2	5.7	[-5.8 ; 17]	0.326	
**Post-NAC parameters**					
**pCR**	**No pCR**				
	**pCR**	**-14.3**	**[-24 ; -5.1]**	**0.003**	**
**Nodal involvment**	ypN-				
	ypN+	3.9	[-6.8 ; 14.6]	0.47	
**LVI**	Absence				
	Presence	3.7	[-13.2 ; 20.6]	0.67	
**SUV at 2 cures**		0.93	[-0.23 ; 2.1]	0.11	
**Variation Pre/Post-NAC parameters**					
**SUV relative variation[b]**				**<0.0001**	***
**SUV relative variation (class)**	**< -70%**				
	**≥ -70%**	**19.5**	**[10 ; 29]**	**<0.0001**	***

[a]continuous variable modelized with a degree 2 polynomial (coefficients and IC : -0.46 [-0.9 ; -0.016] ; -0.007 [-0.013 ; 60.0011]), [b]continuous variable modelized with a degree 2 polynomial (coefficients and IC : -0.5 [-1.2 ; 0.22] ; 60.0084 [-0.015 ; -0.0018]).

Table S4. Predictive factors associated with pCR status.

		**Univariate analyses**				**Multivariate analysis**			
**Parameters**	**Levels**	**OR**	**[95% CI]**	**P-value**		**OR**	**[95% CI]**	**P-value**	
**Pre-NAC parameters**									
Age (class)	<45	1							
	≥45	1.5	[0.65 ; 3.4]	0.36					
Pregnancies	0	1							
	1-3	1.2	[0.51 ; 2.8]	0.67					
	>3	0.98	[0.3 ; 3.2]	0.97					
Menopausal status	premenopausal	1							
	postmenopausal	0.93	[0.44 ; 2]	0.84					
Family history	0	1							
	1	0.99	[0.37 ; 2.6]	0.99					
	≥2	0.67	[0.18 ; 2.4]	0.54					
Clinical tumor stage	T1-T2	1							
	T3	0.49	[0.23 ; 1]	0.064	.				
Clinical nodal status	N-	1							
	N+	0.7	[0.33 ; 1.5]	0.34					
Tumor grade	2	1							
	3	3.2	[0.99 ; 10.3]	0.053	.				
LVI	Absence	1							
	Presence	0.62	[0.21 ; 1.8]	0.38					
TIL levels (continuous)		1	[1 ; 1]	0.11					
Chemotherapy regimen	SIM-1	1							
	SIM-2	0.77	[0.32 ; 1.9]	0.56					
**Post-NAC parameters**									
**TIL levels (continuous)[a]**		**0.29**	**[0.13 ; 0.64]**	**0.002**	**	0.38	[0.17 ; 0.87]	0.023	*
**SUV relative variation**		**0.93**	**[0.91 ; 0.96]**	**<0.0001**	***				
**SUV relative variation (class)**	**< -70%**	**1**				**1**			
	**≥ -70%**	**0.06**	**[0.022 ; 0.16]**	**<0.0001**	***	**0.076**	**[0.027 ; 0.22]**	**<0.0001**	***
**TILs absolute variation**	**<0**	**1**							
	**≥0**	**0.15**	**[0.053 ; 0.44]**	**0.0007**	***				

[a] this OR corresponds to difference of 10 units of the variable (for example, the univariate analysis reveals that a patient with post-NAC TIL levels of 15% will have 3.4 (=1/0.29) times the risk of a non pCR than that of a patient with a level of 5%).

Table S5. Predictive factors associated with overall survival.

		**Univariate analyses**				**Multivariate analysis**			
**Parameters**	**Levels**	**HR**	**[95% CI]**	**P-value**		**HR**	**[95% CI]**	**P-value**	
**Pre-NAC parameters**									
Age (class)	<45	1							
	≥45	0.53	[0.2 ; 1.4]	0.21					
Pregnancies	0	1							
	1-3	0.72	[0.25 ; 2.1]	0.54					
	>3	0.75	[0.15 ; 3.7]	0.73					
Menopausal status	premenopausal	1							
	postmenopausal	0.33	[0.093 ; 1.2]	0.082	.				
Family history	0	1							
	1	0.8	[0.18 ; 3.6]	0.78					
	≥2	2.6	[0.71 ; 9.2]	0.15					
Clinical tumor stage	T1-T2	1							
	T3	1.7	[0.63 ; 4.8]	0.28					
Clinical nodal status	N-	1							
	N+	2.1	[0.73 ; 6.1]	0.17					
Tumor grade	2	1							
	3	2.2	[0.29 ; 16.9]	0.44					
LVI	Absence	1							
	Presence	2.3	[0.73 ; 7.4]	0.15					
TIL levels (continuous)		0.98	[0.95 ; 1]	0.26					
**Post-NAC parameters**									
pCR	No pCR	1							
	pCR	0.4	[0.13 ; 1.3]	0.12					
**Nodal involvment**	**ypN-**	**1**							
	**ypN+**	**3.1**	**[1.2 ; 8.4]**	**0.023**	*				
**LVI**	**Absence**	**1**				**1**			
	**Presence**	**4.4**	**[1.4 ; 13.8]**	**0.011**	*	**4.8**	**[1.5 ; 15.7]**	**0.009**	**
**TIL levels (continuous)[a]**		**1.7**	**[1.3 ; 2.2]**	**0.0001**	***	**1.8**	**[1.3 ; 2.3]**	**<0.0001**	***
**Variation Pre/Post-NAC parameters**									
SUV relative variation		1	[0.99 ; 1]	0.35					
**SUV relative variation (class)**	**< -70%**	**1**							
	**≥ -70%**	**3.2**	**[1 ; 10.2]**	**0.048**	*				
**TILs absolute variation**	**<0**	**1**							
	**≥0**	**3.1**	**[1.1 ; 8.8]**	**0.031**	*				

[a] this HR corresponds to difference of 10 units of the variable (for example, the univariate analysis reveals that a patient with post-NAC TIL levels of 25% will have 1.7 times the risk of death than that of a patient with a level of 15%).

Table S6. Predictive factors associated with overall survival for the Non-pCR subcohort.

		**Univariate analyses**				**Multivariate analysis**		
**Parameters**	**Levels**	**HR**	**[95% CI]**	**P-value**		**HR**	**[95% CI]**	**P-value**
**Pre-NAC parameters**								
Age (factor)	<45	1						
	≥45	0.59	[0.19 ; 1.9]	0.38				
Pregnancies	0	1						
	1-3	1.5	[0.38 ; 5.8]	0.57				
	>3	1.6	[0.26 ; 9.4]	0.63				
Menopausal status	premenopausal	1						
	postmenopausal	0.25	[0.054 ; 1.1]	0.071	.			
Family history	0	1						
	1	0.45	[0.057 ; 3.5]	0.45				
	≥2	1.6	[0.35 ; 7.6]	0.53				
Clinical tumor stage	T1-T2	1						
	T3	1.6	[0.48 ; 5.3]	0.45				

Clinical nodal status	N-	1							
	N+	1.7	[0.53 ; 5.8]	0.36					
LVI	Absence	1							
	Presence	2.4	[0.65 ; 9.1]	0.18					
TIL levels		1	[0.97 ; 1]	0.90					
**Post-NAC parameters**									
Nodal involvment	ypN-	1							
	ypN+	2.5	[0.69 ; 9.4]	0.16					
**LVI**	**Absence**	**1**				**1**			
	**Presence**	**3.6**	**[1 ; 12.2]**	**0.042**	*	**5.5**	**[1.4 ; 22.1]**	**0.016**	*
**TIL levels (continuous)[a]**		**1.8**	**[1.3 ; 2.4]**	**0.0003**	***	**2**	**[1.4 ; 2.8]**	**0.0001**	***
**Variation Pre/Post-NAC parameters**									
SUV relative variation		1	[0.98 ; 1]	0.74					
SUV relative variation (factor)	< -70%	1							
	≥ -70%	3.9	[0.49 ; 30.8]	0.20					
**TILs absolute variation**	**<0**	**1**							
	**≥0**	**3.8**	**[1 ; 14.3]**	**0.048**	*				

[a] this HR corresponds to difference of 10 units of the variable (for example, the univariate analysis reveals that a patient with post-NAC TIL levels of 20% will have 1.8 times the risk of death than that of a patient with a level of 10%).
